# Expression of SOX2, NANOG and OCT4 in a mouse model of lipopolysaccharide-induced acute uterine injury and intrauterine adhesions

**DOI:** 10.1186/s12958-017-0234-9

**Published:** 2017-03-03

**Authors:** Li Xiao, Yong Song, Wei Huang, Shiyuan Yang, Jing Fu, Xue Feng, Min Zhou

**Affiliations:** 1 Department of Obstetrics and Gynecology, West China Second University Hospital of Sichuan University, Chengdu, 610041 Sichuan People’s Republic of China; 2 Key Laboratory of Birth Defects and Related Diseases of Women and Children (Sichuan University), Ministry of Education, Chengdu, 610041 Sichuan People’s Republic of China

**Keywords:** Acute inflammation, Intrauterine adhesions, Sex-determining Y-box2, Nanog homebox, Octamer-binding protein

## Abstract

**Background:**

Activation of inflammation-mediated endometrial injury is suggested to play a decisive role in pathogenesis of intrauterine adhesion (IUA). The stem cell theory of endometrial diseases has been given a hotspot, in that human endometrial stem cells have been isolated from the endometrium. Three transcription factors that play key roles in maintaining pluripotency and self-renewal in stem cells are sex-determining region Y-box2 (SOX2), Nanog homebox (NANOG), and octamer-binding protein (OCT4), which may be responsible for the damage or repair process of uterine endometrium. We aim to investigate the expression of SOX2, NANOG and OCT4 in a mouse model of acute uterine injury induced by peritoneal injection of lipopolysaccharide (LPS) and also analyze their changes in endometrium of women with IUA.

**Methods:**

The mouse uterine horns were collected at 0 h, 6 h, 12 h, 18 h or 24 h after a single dose of LPS or PBS injection. Meanwhile, we recruited 19 women with IUA diagnosed by hysteroscopy and 16 disease-free women as control group. Endometrial tissue samples were collected. SOX2, NANOG, and OCT4 expression were analyzed with Quantitative Real-time Polymerase Chain Reaction and Western blotting assay.

**Results:**

In a mouse model of acute uterine injury, there was significant upregulation of NANOG at 6 h, SOX2 and OCT4 at 12 h compared with the values before injection or PBS injection. NANOG expression reached a peak at 6 h, while SOX2 and OCT4 peaked later at 12 h after LPS treatment. NANOG mRNA and protein expressions were significantly higher in endometrium of IUA patients compared to those of the control group.

**Conclusions:**

Expression of pluripotency factors SOX2, NANOG and OCT4 increased in a mouse model of LPS-induced acute uterine injury. NANOG peaked earlier followed by the other two factors before returning to baseline levels. NANOG but not SOX2 and OCT4 expression was overexpressed in the endometrium of women with IUA. They may be involved in the formation or restoration of IUA, and their roles in pathogenesis of IUA need to be further studied.

## Background

Intrauterine adhesion (IUA) comprises a less severe condition involving partial replacement of endometrial surfaces with fibrotic tissue. Presenting symptoms are related to the degree and location of IUA, and include menstrual abnormalities ranging from irregular bleeding to hypomenorrhea and amenorrhea, infertility and/or recurrent pregnancy loss [1]. Normal endometrial growth is essential for embryo implantation and maintenance of pregnancy. IUA occurs most commonly as a result of trauma or infection, particularly after pregnancy when estradiol levels are low. Infection and inflammation may contribute to the inability of traumatized endometrium to regenerate and are important processes involved in the deposition of fibrotic tissue [2].

Recently, a population of epithelial progenitor cells and mesenchymal stem cells (MSCs) were identified in human endometrium [3]. They are proposed to be responsible for regenerating the functional layer of the endometrium following menstruation and parturition [4]. Human MSCs are used in clinical trials as cell source for tissue regenerative therapy. Recently, they are known to have therapeutic benefits by regulating the immune system invoked in settings such as tissue injury and autoimmunity. MSCs suppress the immune response by several different means, for instance, by secreting anti-inflammatory cytokines such as IL-10 and TGF-β and the inhibition of pro-inflammatory cytokines such as IL-6 and TNF-α [5–7]. Single or repeated biopsy of the thin endometrium induce an injury response that stimulates resident endometrial stem/progenitor cells to initiate a regenerative response and restore endometrial tissue homeostasis [2]. Human endometrial Side Population (SP) cells, a component of putative progenitor/stem cells, rapidly accumulated in mice endometrium during acute uterine injury induced by lipopolysaccharide (LPS) [8]. Though the acute inflammatory response may be beneficial to the endometrium, in IUA the inflammation is self-perpetuating and persists for a long period and can trigger abnormal endometrial functions to facilitate the formation of fibrosis and adhesion.

However, with lack of specific surface markers for endometrial stem cells and the technical restrictions on endometrial tissue samplings, researches on mesenchymal stem/progenitor cells in endometrial diseases, especially in IUA, are scarce. Related studies mainly focus on stem-ness related genes. Increasing evidence suggests that three transcription factors: sex-determining region Y-box 2 (SOX2), Nanog homeobox (NANOG), and octamer-binding protein 4 (OCT4)are most frequently involved in endometrial diseases [9–11]. SOX2, NANOG and OCT4 cooperatively maintain the regulatory network and play a pivotal role in maintaining the pluripotency and self-renewal of stem cells [12]. They co-occupy and regulate many developmentally important home domain genes and collaborate to form an extensive regulatory circuitry including auto-regulatory and feed-forward loops [13]. However, their role in acute uterine injury, as well as in women with IUA have not been identified. Therefore, we aimed to investigate the changes in expression of SOX2, NANOG, and OCT4 using a mouse model of acute uterine injury induced by peritoneal LPS injection. We also investigated these stem-ness related genes in the endometrium of reproductive-age women with or without IUA.

## Methods

### Uterine acute inflammation mouse model

This study was carried out in strict accordance with the recommendations of the Guide for the Care and Use of Laboratory Animals of the National Institutes of Health. The protocol was approved by the Ethics Committee of West China University Hospital of Sichuan University. Female BALB/c mice aged 8–16 weeks were purchased from Chengdu Dashuo Experimental Animals Limited Company (Chengdu, Sichuan, China). All animals were housed in filter-top cages with autoclaved bedding, autoclaved food and water ad libitum, and a 12 h light/dark cycle.

BALB/c mice were grouped into two: Control and LPS injected groups. A single dose of. LPS (0.5 mg/kg, Sigma-Aldrich, St. Louis, MO, USA) was injected intraperitoneally to BALB/c mice to cause endometrial injury [14]. Control mice were injected with an equal volume of PBS. Mice were sacrificed by cervical dislocation at 6, 12, 18, 24 h and uterine horns were collected. Part of specimens derived from LPS-treated and control mice were fixed in formaldehyde and embedded in paraffin. 5 μm sections were cut and stained with hematoxylin and eosin (H&E). Other Specimens were used for Quantitative Real-time Polymerase Chain Reaction (qRT-PCR) and Western blotting.

### Human study population

The Ethical Committee of West China Second University Hospital of Sichuan University approved the study and informed consent was obtained from each participant. Women aged ≤40 years with no hormonal treatment for at least 3 months before surgery were included in the IUA group. Similarly, women aged ≤40 years with regular menstrual cycles and not taking hormonal treatment 3 months before surgery and with no evidence of intrauterine adhesions were included in the control group, The exclusion criteria included endometriosis, adenomyosis, leiomyomas, hydrosalpinx, endometrial polyp, endometrial hyperplasia, and acute pelvic inflammatory diseases. From March 2014 to October 2014, we recruited 35 women who had undergone simultaneous hysteroscopy and laparoscopy for this study. Of these, 19 women with intrauterine adhesions were diagnosed by hysteroscopy and further confirmed by pathology. The r-AFS intrauterine adhesion score standard was used for disease stage (11 stage mild and 8 stage moderate) [15]. The remaining 16 were controls that had undergone simultaneous laparoscopy and hysteroscopy for infertility that had no visible evidence of intrauterine adhesions during surgery (Table 1). Endometrial tissues were obtained by scraping the endometrium with a uterine curette during hysteroscopic procedure from the adhesion sites of IUA and control patients. Of the 19 IUA specimens, 13 were used for qRT-PCR and remaining six for Western blotting. Of the 16 control specimens, 11 were used for qRT-PCR and remaining five for Western blotting. Tissues were stored in a microfuge tube at −80 °C for qRT-PCR and Western blotting analysis, or immediately fixed in 10% buffered formalin and paraffin-embedded for H&E staining. All participants were determined to be in the proliferative phase of their menstrual cycle as assessed by the timing of their last menstrual period and histological examination.

**Table 1:** Characteristics of study population

Characteristics	IUA group	Non-IUA group
Number of cases (n)	19	16
Age (yrs.)	31.69 ± 4.35 (25 ~ 40)	29.27 ± 4.74 (23 ~ 36)
Infertility duration (yrs.)	3.32 ± 1.73 (1 ~ 8)	3.00 ± 1.21 (1 ~ 5)
Adhesion staging (n)		
Mild	11	0
Moderate	8	0

### RNA extraction, reverse transcription, and quantitative real-time polymerase chain reaction

All the tissue samples were frozen in liquid nitrogen immediately after collection. Total RNA was extracted using TRIzol, according to the manufacturer’s instructions (Life Technologies Inc., Carlsbad, CA, USA) and, the quality and concentration of purified RNA were analyzed using Nano Drop Plus spectrophotometer (Health-careBio-Science AB, Uppsala, Sweden). The nucleotide to protein ratios (A260:A280) of all samples were within the acceptable boundaries of 1.8 and 2.1. First-strand complementary DNA (cDNA) was synthesized using Prime Scriptreverse transcription (RT) Reagent Kit (TaKaRa Biotechnology, Dalian, China) at 37 °C for 15 min, followed by deactivation at 85 °C for 5 s, according to the manufacturer’s protocol. The primer sequences were synthesized by Sangon Biotech (Shanghai, China) and described in Table 2. cDNA was amplified using EvaGreen Supermix (Bio-Rad Laboratories, Hercules, CA, USA). Amplification was performed by initial denaturation at 95 °C for 30 s followed by 40 cycles of denaturation at 95 °C for 5 s and annealing/elongation at 60 °C for 10 s. Melting curve analysis confirmed the amplification of a single product. The qRT-PCR assays were performed in duplicate in three independent experiments for each experimental condition. Human or mouse glyceraldehyde-3-phosphate dehydrogenase (*GADPH*) was used for normalization of the qRT-PCR results.

**Table 2:** Primer sequences used in quantitative real-time PCR in human and mouse specimen

Gene	Sense primer 5’ –3’	Antisense primer 5’ –3’
*GAPDH (human)*	TGCACCACCAACTGCTTAGC	GGCATGGACTGTGGTGATGAG
*SOX2 (human)*	TACAGCATGTCCTACTCGCAG	GAGGAAGAGGTAACCACAGGG
*NANOG (human)*	AAGGTCCCGGTCAAGAAACAG	CTTCTGCGTCACACCATTGC
*OCT4 (human)*	GCAGCGACTATGCACAACGA	CCAGAGTGGTGACGGAGACA
*GAPDH (mouse)*	TGACCTCAACTACATGGTCTACA	CTTCCCATTCTCGGCCTTG
*SOX2 (mouse)*	GCGGAGTGGAAACTTTTGTCC	GGGAAGCGTGTACTTATCCTTCT
*NANOG (mouse)*	CACAGTTTGCCTAGTTCTGAGG	GCAAGAATAGTTCTCGGGATGAA
*OCT4 (mouse)*	CGGAAGAGAAAGCGAACTAGC	ATTGGCGATGTGAGTGATCTG

### Western blotting

Total protein was collected using radio immunoprecipitation lysis buffer (P0013B, Beyotime Biotechnology, Shanghai, China) according to the manufacturer’s instructions. Protein concentration was determined using a bicinchoninic acid assay kit (Beyotime Biotechnology). Protein (30 μg) were separated using 10% sodium dodecyl sulfate–polyacrylamide gel electrophoresis and transferred to polyvinylidene fluoride membranes (Millipore, Billerica, MA, USA). The membranes were blocked for 1 h in 5% defatted milk at room temperature. Subsequently, the membranes were incubated with monoclonal mouse anti-human SOX2 (1:400; ab75485, Abcam, Cambridge, UK), polyclonal rabbit anti-human NANOG (1:400; ab80892, Abcam), OCT4 (1:800; ab18976, Abcam), and polyclonal mouse anti-β-actin (1:30000; bs-2188R, Bioss, Beijing, China) antibodies overnight at 4 °C. Then the membranes were incubated with horseradish peroxidase–conjugated secondary anti-mouse/rabbit antibody for 1 h at room temperature. Proteins bands were visualized using an enhanced chemiluminescence system (Millipore) and analyzed with ImageJ 2x (National Institutes of Health, Bethesda, MD, USA). Protein levels were normalized to that of the internal control β-actin.

### Statistical analysis

Statistical analysis was performed using SPSS version 18.0 (SPSS, Chicago, IL, USA). All data are expressed as means ± SEM. Differences between groups were analyzed with one-way analysis of variance followed by a post hoc test (Student–Newman–Keuls method). Statistical significance was determined as *P* <0.05 (two-tailed).

## Results

### LPS induces uterine inflammation

**Fig. 1:**
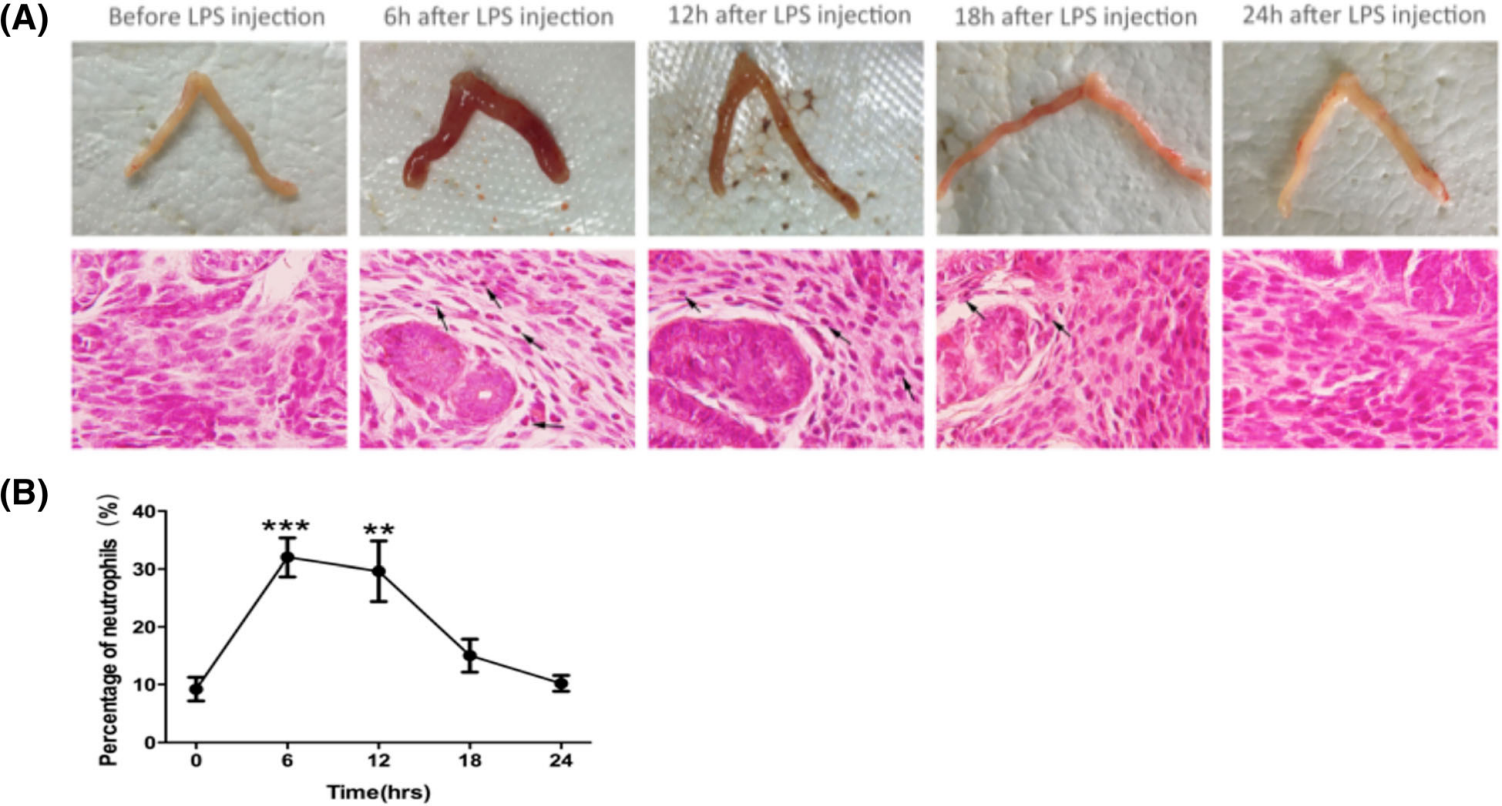
LPS-induced uterine endometrial injury. **a** The gross uterine specimen and histological finding of uterine endometrium. Sections of uterine endometrium were obtained and stained with H&E. Edema and neutrophils (arrows) were observed in the endometrial epithelium and stroma. **b** The percentage of neutrophils in the uterine endometrium. The percentage increased significantly in mice uterine and peaked at 6 h after LPS injection. Values are represented as mean ± SEM. ^★★★^ *P* <0.001 vs.0 h

**Fig. 2:**
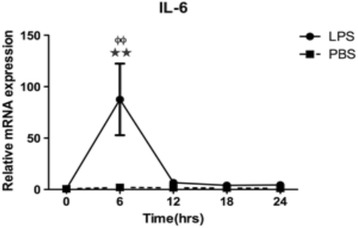
Changes of *IL-*6 mRNA expression in mice injured uterine injected with LPS or PBS (control). Analysis was carried out on mice uterine before injection, after LPS or PBS injection. The expression of *IL*-6 mRNA increased significantly in mice uterine and peaked at 6 h after LPS injection. Values are represented as mean ± SEM. ^★★^ *P* <0.005 vs.0 h, ^ɸɸ^ *P* <0.005vs. PBS

Uterine endometrial injury was observed at 6–12 h after LPS injection (Fig. 1a). It was characterized by the presence of edema macroscopically. and neutrophils (arrows) in the uterine endometrial epithelium and stroma 6 h after LPS injection. Repair of the endometrial injury was evident by 24 h after LPS injection. The neutrophils were significantly increased 6 h after LPS injection (*P* = 0.000 vs. 0 h), and returned to normal level before the injection 24 h after injection (Fig. 1b). Interleukin-6 (IL-6) mRNA expression in mice uterine was significantly increased and reached a peak at 6 h after LPS injection (*P* = 0.002 vs. 0 h). Expression of IL-6 mRNA decreased rapidly at 12 h, and remained at a low level at 18 h and 24 h after LPS injection (*P* = 0.812, 0.898, 0.888 vs. 0 h), which are not significantly different compared to that before LPS or PBS injection, hence the inflammatory reaction was not significant since then (Fig. 2).

### Expression of *SOX2*, *NANOG*, and *OCT4* mRNA in LPS-induced acute uterine injury

**Fig. 3:**
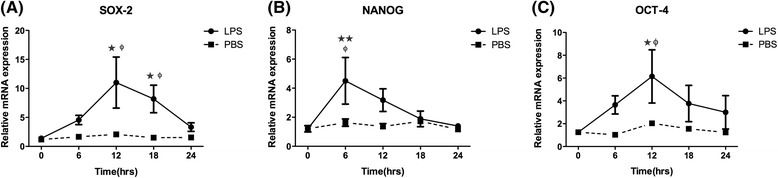
qRT-PCR analysis of *SOX2*, *NANOG*, and *OCT4* mRNA in mice injured uterine injected with LPS or PBS (control). **a**-**c**) Analysis was carried out on mice uterine before injection (*n* = 5), after LPS (6 h, *n* = 6; 12 h, *n* = 6; 18 h, *n* = 5; 24 h, *n* = 5) or PBS (6 h, *n* = 4; 12 h, *n* = 4; 18 h, *n* = 4; 24 h, *n* = 4) injection. Values are represented as mean ± SEM. ^★★^ *P* <0.005 vs.0 h, ^★^ *P* <0.05 vs.0 h, ^ɸ^ *P* <0.05vs. PBS

The *NANOG* mRNA sharply increased, peaking at 6 h after LPS injection significantly (*P* = 0.02 vs. 0 h, *P* = 0.000 vs PBS). Then, the expression gradually decreased at 12, 18, and 24 h after LPS injection. The expression of *SOX2* mRNA also increased in the uterus and reached a peak at 12 h after LPS injection. This was significantly higher at 12 h and 18 h (*P* = 0.01 vs. 0 h) and returned to baseline after 24 h after LPS injection. Similar to the expression of *SOX2* mRNA, *OCT4* mRNA increased after LPS injection, and reached a peak at 12 h after LPS injection. There was a significant difference only at 12 h after LPS injection and returned to baseline after 24 h (*P* = 0.04 vs. 0 h) (Fig. 3).

### Expression of SOX2, NANOG, and OCT4 proteins in LPS-induced injured uterus

**Fig. 4:**
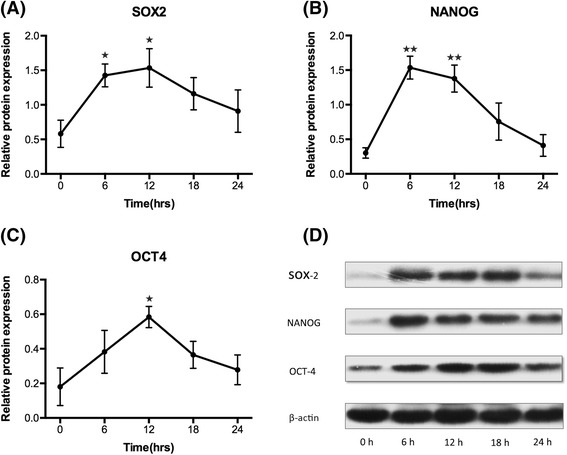
Western Blotting analysis of SOX2, NANOG, and OCT4 protein in mice injured uterine injected with LPS. **a**-**c** Analysis was carried out on mice uterine before injection (*n* = 3) and after LPS injection (6 h, *n* = 4; 12 h, *n* = 4; 18 h, *n* = 3; 24 h, *n* = 3). Values are represented as mean ± SEM. ^★★^ *P* <0.005 vs.0 h, ^★^ *P* <0.05 vs.0 h. **d** Representative western blotting of SOX2, NANOG, and OCT4 protein. β-actin, internal control

Western blotting demonstrated that the expression of SOX2, NANOG, and OCT4 protein all increased after LPS injection. Similar to the qPCR results, the expression of NANOG protein peaked at 6 h, SOX2 and OCT4 protein peaked at 12 h after LPS injection, while after LPS injection. LPS injection led to a significant increase in SOX2 protein in mice uterine at 6 h, 12 h after LPS injection (*P* = 0.04, *P* = 0.04 vs. 0 h), NANOG protein in mice uterine at 6 h, 12 h after LPS injection (*P* = 0.001, *P* = 0.001vs. 0 h) and OCT4 protein at 12 h after LPS injection (*P* = 0.02 vs. 0 h) (Fig. 4).

### SOX2, NANOG, and OCT4 mRNA and protein expression in the endometrium of women with IUA

**Fig. 5:**
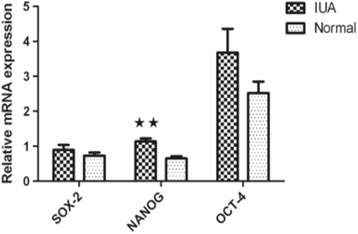
qRT-PCR analysis of *SOX2*, *NANOG*, and *OCT4* mRNA in IUA or normal endometrium. Analysis was carried out on normal endometrium (*n* = 11) and IUA endometrium specimens (*n* = 13). ^★★^ *P* <0.05. Values are represented as mean ± SEM, Error bars denote SEM

**Fig. 6:**
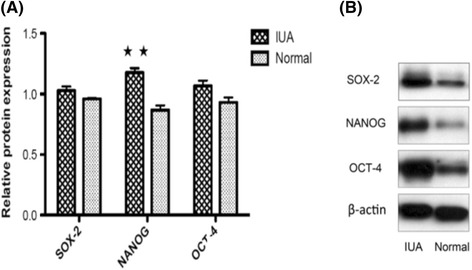
Western blotting analysis of SOX2, NANOG, and OCT4 protein in IUA or normal endometrium. **a** Analysis was carried out on normal endometrium (*n* = 5) and IUA endometrium specimens (*n* = 6). ^★★^ *P* <0.05. Values are represented as mean ± SEM; Error bars denote SEM. **b** Representative Western Blotting of SOX2, NANOG, and OCT4 protein. β-actin, internal control

*NANOG* mRNA expression in the endometrium of IUA women was significantly higher than that in normal endometrium (*P* = 0.008). No significant differences were observed in the expression of *SOX2* and *OCT4* genes in the IUA endometrium compared to the normal endometrium (*P* >0.05) (Fig. 5). Similarly, NANOG protein expression in the endometrium of IUA women was significantly increased compared to the normal endometrium, (*P* = 0.035). SOX2 and OCT4 protein expression in IUA endometrium tended to be higher, but were not statistically significant (*P* >0.05) (Fig. 6).

## Discussion

Murine endometrium consists of a single layer that contains epithelium and stroma [16]. Therefore, the murine endometrium is considered a suitable model of uterine endometrial research. Mounting evidence suggests that the evolutionarily adaptive process of acute inflammation, which is needed for wound-healing or eliminating after uterine curettage or operation, can be maladaptive if it is sustained chronically. Thus, LPS, the major component of endotoxin in gram-negative bacterial infection, can enter the blood stream and elicit systemic inflammatory response with increased production of pro-inflammatory mediators such as IL-6 [17]. We established a mouse model of acute uterine injury to explore expression of stem cell and proteins response to acute inflammation over a 24 h time period to understand the initial acute phase of IUA. To our knowledge, this study provides the first evidence that LPS induced acute inflammation provokes a rapid increase in transcription factor SOX2, NANOG and OCT4 mRNA and protein levels in the uterus. These upregulated changes returned to baseline within 24 h. It is therefore conceivable that tissue injury could induce a temporary increase in SOX2, NANOG and OCT4 at mRNA and protein levels. Upregulation of these transcriptional factors in correspondence with inflammatory response and their levels recovering following subsidence of inflammation suggests that these factors may contribute to the endometrial regeneration following an acute insult.

Taylor el al. have reported that progenitor/stem cells might reside in the uterine endometrium with or without injury and may differentiate into uterine endometrial stroma and epithelium [18]. This is compatible with our findings that stem-ness related genes were present even in the absence of injury, and injury increased transcriptional factor SOX2, NANOG and OCT4 in mouse uterus. A previous study demonstrated that SP cells, a cluster of stem/progenitor cell in adult tissue [19], rapidly accumulated in a mouse model of LPS-induced endometrial injury. Endometrial injury could not only stimulate residence progenitor/stem cells proliferation but also attract and promote migration of bone marrow stem cells to the endometrium [4, 18, 20]. As it is suggested that the progenitor/stem cells could be responsible for the cyclical regeneration of the uterine endometrium [21], and the stem-ness related genes, which play a key role in maintaining the pluripotency and renewable of stem cells, could be also responsible for the rapid regeneration of endometrium during menstruation or injury. NANOG was observed to attenuate inflammatory response of BV-2 cells [22] and rat primary microglial cells [23] stimulated by LPS. It also inhibits the NO release, iNOS expression and pro-inflammatory mediators IL-6, IL-1β and TNF-α, by blocking NF-kB transcriptional activity, that supporting NANOG may be the mechanism for reduction of pro-inflammatory factors. However, SOX2 overexpression upregulated genes associated with tissue damage and inflammation, including IL-1β and IL-6, and it cooperates with inflammatory signaling mediated through the IL-1β/IL-6/Stat3 pathway, to transform progenitor cells [24]. Regardless, our finding of those stem transcription factors expression in mouse model speculates for the first time that the acute injury seems to be the triggering event in mouse uterus that initiate an up-regulation of stem-ness related genes, which may be a potential anti-inflammatory therapy or the pathogenesis of endometrial inflammation related diseases.

On the other hand, It is well known that 90% of genes with promoters bound by OCT4 and SOX2 in human ESCs are also bound by NANOG [25], Originally, we found it intriguingly that an immediate rapid rise in NANOG expression, which reached a peak at 6 h, followed by a rise in SOX2 and OCT4 genes, with peak up-regulation at 12 h after LPS treatment. Therefore, consistent with our data, Chambers and coworkers have also shown that it is possible that NANOG may alleviate the requirement for SOX2 in reprogramming by stimulating or maintaining OCT4 expression. Indeed, NANOG can maintain OCT4 expression in mESCs [26]. It is reported that NANOG expression enhanced the reprogramming of human fibroblasts but that it was not able to replace SOX2 in the presence of only OCT4 and LIN-28 [27]. We have shown that there may be a delay for SOX2 and OCT4 to regulate downstream genes chronologically, following by NANOG, which could be a trigger for other two factors, and the specific mechanism need to be further studied.

In this study, we have endeavored to systematically investigate the expression and origin of transcriptional factors in IUA. The expression of NANOG mRNA and protein, and that of two other core stem cell transcription factors, SOX2 and OCT4, were detected in IUA endometrium [11]. We showed significantly higher expression of *NANOG* mRNA in IUA endometrium as well as NANOG protein, when compared with normal endometrium. The increasing NANOG expression in our study may indicate stem cell origin pathological mechanism of IUA. Recent studies have demonstrated a key role for the NANOG gene in maintaining pluripotency and self-renewal of stem cells [26]. Overexpression of mouse or human NANOG in ES cells can overcome the requirement for leukocyte inhibitor factor to maintain the undifferentiated state and can block differentiation in the presence of retinoic acid or3-methoxybenzamide, while the deletion of NANOG gene resulted in loss of pluripotency in both ICM and ESCs [16, 26]. Compared to the all three transcriptional factors overexpression in the acute inflammatory response, the upset of balance between pro-inflammation and anti-inflammation, mediated by those transcription factors may be the breakthrough of IUA, which only overexpressed NANOG.

When given a single dose of LPS treatment, the related transcription factors and their network may play a role in endometrial inflammation and repair. OCT4 and SOX2 cooperatively bind many genomic sites as heterodimers. NANOG binding also shows extensive overlap with that of OCT4 and SOX2 [28]. However, there may be functional differences between OCT4 and SOX2 modules and the NANOG module, as they also regulated independently different down-stream target genes [26, 29]. In case of IUA with higher levels of NANOG, there is persistent chronic inflammation. The changes of OCT4-SOX2-NANOG occupancy do not correlate well with differential gene expression. Thus, the interconnected network may be broken down, and NANOG function independently leading to stem cells differentiation into myofibroblast and formation of endometrial fibrosis. We believe that this may be a potential mechanism that leads to the intrinsic differentiation of endometrial stem cells, and play a vital role in the pathogenesis of disease, thus supporting the “stem cell” hypothesis of IUA.

The present study has some limitations and unanswered questions that need to be addressed to improve upon future studies. First, sample size is not large and amount of endometrial tissue, especially endometrial tissues from IUA are extremely bare, cannot match all experimental items that may result in some index not reach statistical significance. Second, this study only demonstrated the increased expression of transcription factors. It would be important to confirm the specific mechanism of their roles in IUA, an inflammatory endometrial disease. Studies on larger sample size in future would be helpful in understanding the molecular mechanism of SOX2, NANOG and OCT4 in IUA.

## Conclusions

In acute inflammatory mouse model, the expression of transcriptional factors, SOX2, NANOG and OCT4 in the uterus peaks and levels off to base line after the inflammation subsides. This may be vital response in an acute insult for tissue repair and regeneration. However, only NANOG is overexpressed in the endometrium of reproductive-age women with IUA. This may suggest a mismatch in cross-talk between NANOG with OCT4 and SOX2 in IUA leading to failure/faulty in endometrial repair and replacing with fibrotic tissue. Further studies should be required to identify the specific transcriptional factors’ role in pathogenesis of IUA. However, it will be of significant interest to determine these transcription factors could be involved in the formation or restoration of IUA, as well as in acute uterine injury.

## Abbreviations

ESCs: Embryo stem cells
ICM: Inner cell mass.
IUA: Intrauterine adhesion
LPS: Lipopolysaccharide
MSCs: Mesenchymal stem cells
NANOG: Nanog homebox
OCT4: Octamer-binding protein 4
SOX2: Sex-determining region Y-box 2
SP: Side population

## References

[1] Panayiotides I, Weyers S, Bosteels J, Herendael B (2009). Intrauterine adhesions(IUA): has there been progress in understanding and treatment over the last20 years?. Gynecol Surg.

[2] Gargett CE, Healy DL (2011). Generating receptive endometrium in Asherman’s syndrome. J Hum Reprod Sci.

[3] Schwab KE, Chan RW, Gargett CE (2005). Putative stem cell activity of human epithelial and stromal cells during the menstrual cycle. Fertil Steril.

[4] Gargett CE (2007). Uterine stem cells: what is the evidence?. Hum Reprod Update.

[5] Park MJ, Park HS, Cho ML, Oh HJ, Cho YG, Min SY (2011). Transforming growth factor β-transduced mesenchymal stem cells ameliorate experimental autoimmune arthritis through reciprocal regulation of Treg/Th 17 cells and osteoclastogenesis. Arthritis Rheum.

[6] Pluchino S, Zanotti L, Brambilla E, Rovere-Querini P, Capobianco A, Alfaro-Cervello C (2009). Immune regulatory neural stem/precursor cells protect from central nervous system autoimmunity by restraining dendritic cell function. PLoS One.

[7] Rajasingh J, Thangavel J, Siddiqui MR, Gomes I, Gao XP, Kishore R (2011). Improvement of cardiac function in mouse myocardial infarction after transplantation of epigenetically-modified bone marrow progenitor cells. PLoS One.

[8] Hyodo S, Matsubara K, Kameda K, Matsubara Y (2011). Endometrial injury increases side population cells in the uterine endometrium: a decisive role of estrogen. Tohoku J Exp Med.

[9] Forte A, Schettino MT, Finicelli M, Cipollaro M, Colacurci N, Cobellis L (2009). Expression pattern of stemness-related genes in humanendometrial and endometriotic tissues. Mol Med.

[10] Chang JH, Au HK, Lee WC, Chi CC, Ling TY, Wang LM (2013). Expression of the pluripotent transcription factor OCT4 promotes cell migration in endometriosis. Fertil Steril.

[11] Song Y, Xiao L, Fu J, Huang W, Wang Q, Zhang X, Yang S (2014). Increased expression of the pluripotency markers sex-determining region Y-box 2 and Nanog homeobox in ovarian endometriosis. Reprod Biol Endocrinol.

[12] Jaenisch R, Young R (2008). Stem cells, the molecular circuitry of pluripotency and nuclear reprogramming. Cell.

[13] Xu RH, Sampsell-Barron TL, Gu F, Root S, Peck RM, Pan G (2008). NANOG is a direct target of TGF beta/activin-mediated SMAD signaling in human ESCs. Cell Stem Cell.

[14] Fischetti F, Carretta R, Borotto G, Durigutto P, Bulla R, Meroni PL (2004). Fluvastatin treatment inhibits leucocyte adhesion and extravasation in models of complement-mediated acute inflammation. Clin Exp Immunol.

[15] Buttram V, Gomel V, Siegler A, DeCherney A, Gibbons W, March C (1988). The american fertility society classifications of adnexal adhesions, distal tubal occlusion, tubal occlusion secondary to tubal ligation, tubal pregnancies, mullerian anomalies and intrauterine adhesions. Fertil Steril.

[16] Martin L, Finn C, Trinder G (1973). Hypertrophy and hyperplasia in the mouse uterus after ostrogen treatment: an autoradiographic study. J Endocrinol.

[17] Ogura N, Matsuda U, Tanaka F, Shibata Y, Takiguchi H, Abiko Y (1996). In vitro senescence enhances IL-6 production in human gingival fibroblasts induced by lipopolysaccharide from campylobacterrectus. Mech Ageing Dev.

[18] Taylor HS (2004). Endometrial cells derived from donor stem cells in bone marrow transplant recipients. JAMA.

[19] Kato K, Yoshimoto M, Kato K, Adachi S, Yamayoshi A, Arima T (2007). Characterization of side-population cells in human normal endometrium. Hum Reprod.

[20] Tibbetts TA, Conneely OM, O’Malley BW (1999). Progesterone via its receptor antagonizes the pro-inflammatory activity of estrogen in the mouse uterus. Biol Reprod.

[21] Gargett CE, Chan RW (2006). Endometrial stem/progenitor cells and proliferative disorders of the endometrium. Minerva Ginecol.

[22] Duan Z, Ma C, Han Y, Li Y, Zhou H (2013). Nanog attenuates lipopolysaccharide-induced inflammatory responses by blocking nuclear factor-κB transcriptional activity in BV-2 cells. Neuro Rep.

[23] Zhou H, Chen S, Wang W, Wang Z, Wu X, Zhang Z (2012). Nanog inhibits lipopolysaccharide-induced expression of pro-inflammatory cytokines by blocking NF-κB transcriptional activity in rat primary microglial cells. Mol Med Rep.

[24] Liu K, Jiang M, Lu Y, Chen H, Sun J, Wu S (2013). Sox2 cooperates with inflammation-mediated stat3 activation in the malignant transformation of foregut basal progenitor cells. Cell Stem Cell.

[25] Boyer LA, Lee TI, Cole MF, Johnstone SE, Levine SS, Zucker JP (2005). Core transcriptional regulatory circuitry in human embryonic stem cells. Cell.

[26] Chambers I, Colby D, Robertson M, Nichols J, Lee S, Tweedie S (2003). Functional expression cloning of nanog, a pluripotency-sustaining factor in embryonic stem cells. Cell.

[27] Yu J, Vodyanik MA, Smuga-Otto K, Antosiewicz-Bourget J, Frane JL, Tian S (2007). Induced pluripotent stem cell lines derived from human somatic cells. Science.

[28] Chen X, Xu H, Yuan P, Fang F, Huss M, Vega VB (2008). Integration of external signaling pathways with the core transcriptional network in embryonic stem cells. Cell.

[29] Chew JL, Loh YH, Zhang W, Chen X, Tam WL, Yeap LS (2005). Reciprocal transcriptional regulation of Pou5f1 and Sox2 via the Oct4/Sox2 complex in embryonic stem cells. Mol Cell Biol.

